# *Baylisascaris procyonis*–Associated Meningoencephalitis in a Previously Healthy Adult, California, USA

**DOI:** 10.3201/eid2208.151939

**Published:** 2016-08

**Authors:** Charles Langelier, Michael J. Reid, Cathra Halabi, Natalie Witek, Alejandro LaRiviere, Maulik Shah, Michael R. Wilson, Peter Chin-Hong, Vanja Douglas, Kevin R. Kazacos, Jennifer M. Babik

**Affiliations:** University of California, San Francisco, California, USA (C. Langelier, M.J. Reid, C. Halabi, N. Witek, A. LaRiviere, M. Shah, M.R. Wilson, P. Chin-Hong, V. Douglas, J.M. Babik);; Purdue University College of Veterinary Medicine, West Lafayette, Indiana, USA (K.R. Kazacos)

**Keywords:** Baylisascaris procyonis, baylisascariasis, eosinophilic meningitis, raccoon, roundworm, nematode, parasite, intestinal parasite, encephalitis, meningitis, meningoencephalitis, eosinophilia, zoonoses, helminths, United States, California

## Abstract

After severe neurocognitive decline developed in an otherwise healthy 63-year-old man, brain magnetic resonance imaging showed eosinophilic meningoencephalitis and enhancing lesions. The patient tested positive for antibodies to *Baylisascaris* spp. roundworms, was treated with albendazole and dexamethasone, and showed improvement after 3 months. Baylisascariasis should be considered for all patients with eosinophilic meningitis.

Over the past 30 years, the raccoon-associated roundworm *Baylisascaris procyonis* has emerged as an uncommon but noteworthy human pathogen associated with devastating eosinophilic meningoencephalitis in 25 patients (*1*–*4*). We report a case of neural larva migrans in an otherwise generally healthy man in California, USA.

## Case Report

**Figure 1:**
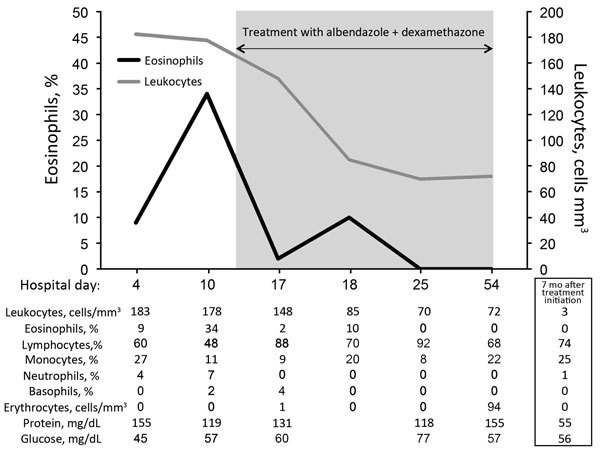
Cell counts and laboratory values in cerebrospinal fluid from a previously healthy adult with *Baylisascaris* meningoencephalitis, California, USA. Hospital day 4 was June 1, 2015; hospital day 54 was July 25, 2015. Samples for 7-month values were obtained on January 1, 2016.

On May 18, 2015, a 63-year-old man was hospitalized in Humboldt County, California, after 2 weeks of fatigue, memory impairment, and progressive confusion accompanied by right-sided occipital headache and right-sided allodynia involving his arm and head. He was confused and disoriented to date but could recognize family; engage in brief, logical conversations; and walk independently. His medical history included essential thrombocytosis, hypothyroidism, and a remote episode of shingles. Vital signs were normal; physical examination showed no focal abnormalities. His complete blood count showed a leukocyte count of 11.5 × 10^9^ cells/L (reference range 3.4–10 × 10^9^ cells/L), eosinophil count of 0.75 × 10^9^ cells/L (reference range <0.4 × 10^9^ cells/L), and neutrophil count of 6.1 × 10^9^/L (reference range 1.8–6.8 × 10^9^ cells/L). Chemistry and liver panel results were normal. A brain magnetic resonance imaging (MRI) demonstrated no intracranial pathology. Cerebrospinal fluid (CSF) showed a leukocyte count of 183 × 10^9^ cells/L (60% lymphocytes, 27% monocytes, 9% eosinophils, 4% neutrophils); protein level of 155 mg/dL; and glucose level of 45 mg/dL (Figure 1). He was started on empiric vancomycin, ceftriaxone, and acyclovir.

Over the next 3 days, the patient sustained precipitous cognitive and functional declines; incontinence, right-sided facial droop, dysarthria, diffuse hyperreflexia, and ataxia developed. Initial infectious disease diagnostics returned negative results (Table 1), so antimicrobial drugs were discontinued. CSF analysis on day 11 showed persistent pleocytosis and marked elevation of eosinophils to 34% (Figure 1). CSF cytologic and flow cytometric testing showed no malignant cells but did show reactive lymphocytes and many eosinophils, consistent with chronic inflammation.

**Table 1:** Microbiologic diagnostics obtained during testing of a previously healthy patient with *Baylisascaris* meningoencephalitis, California, USA*

Diagnostic study	Site	Result
Bacterial cultures ×4	Blood and CSF	Negative
*Coxiella* antibody	Blood	Negative
*Bartonella henselae* and *B. quintana* antibodies	Blood	Negative
*Mycoplasma* antibody	Blood	IgM negative, IgG 1:5
Rickettsial antibody panel	Blood	Negative
Venereal Disease Research Laboratory test	CSF	Negative
Lyme disease antibody	CSF	Negative
Cytomegalovirus PCR	CSF	Negative
Epstein–Barr virus PCR	CSF	Negative
Enterovirus PCR	CSF	Negative
Herpes simplex virus PCR	CSF	Negative
Lymphocytic choriomeningitis virus IgM, IgG	CSF	IgM 1:2, IgG negative†
Varicella zoster virus PCR, IgM, IgG	CSF	Negative
West Nile virus IgM, IgG	CSF	Negative
*Baylisascaris* antibody	Blood and CSF	Positive
*Strongyloides* antibody	Blood	Negative
*Trichinella* antibody	Blood	Negative
*Toxocara* antibody	Blood	Negative
*Toxoplasma* antibody	Blood	Negative
Ova and parasite stain	CSF	Negative
Fungal stains and cultures ×4	Blood and CSF	Negative
*Coccidiodes* antibody by complement fixation	Blood and CSF	Negative
*Coccidiodes* antibody by immunodiffusion	Blood	Negative
Cryptococcal antigen	Blood and CSF	Negative
AFB stains and cultures ×4	CSF	Negative
Broad-range PCR (bacteria, fungi, AFB)	CSF	Negative
Cytology	CSF	Chronic inflammation

*AFB, acid-fast bacilli; CSF, cerebrospinal fluid.  †A low-titer IgM for lymphocytic choriomeningitis virus was considered to be a false-positive result.

**Figure 2:**
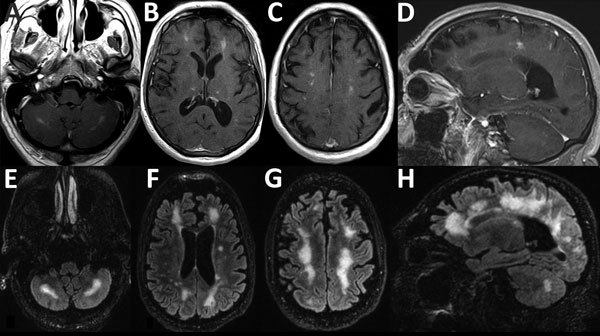
Magnetic resonance imaging scans showing brain abnormalities in a previously healthy adult with *Baylisascaris* meningoencephalitis, California, USA. A–D) Postgadolinium contrast T1 images obtained 4 weeks after symptom onset. A–C) Axial images, moving inferiorly to superiorly, demonstrating nodular bilateral enhancement within the cerebellar hemispheres, thalami, and subcortical white matter. D) Sagittal image further demonstrates multifocal areas of enhancement in cerebral hemispheres. Additional, mild T2 abnormalities (not shown) were present at the same time. E–H) T2/FLAIR (fluid attenuation inversion recovery) images obtained 6 weeks after symptom onset. E–G) Axial images, moving inferiorly to superiorly, demonstrating patchy and confluent hyperintense lesions throughout the supratentorial white matter and cerebellum. H) Sagittal image further demonstrates the high degree of white matter abnormality, which was not present on the earlier imaging. Postcontrast enhancement on T1 imaging (not shown) had nearly resolved at this time.

A brain MRI on day 13 showed new nodular enhancement at the gray–white junction (Figure 2, panels A–D) and patchy T2 signal abnormalities in the cerebellar, pontine, and supratentorial white matter. Due to progressive severe functional and cognitive decline, an unclear diagnosis, and concerning MRI abnormalities, the patient was transferred on day 15 to the University of California San Francisco Medical Center for evaluation. Upon transfer, he was lethargic and had moderate global aphasia and echolalia, a left forehead–sparing facial droop, spasticity in the arms, diffuse hyperreflexia, and mute plantar responses. 

Additional history from his family revealed that the patient had worked as a contractor for >40 years in northern California. Several weeks before symptom onset, he had completed a project under his house, where raccoons and a skunk had been observed, and he had spent significant time working in a rural area with suspected raccoon activity. His occupation necessitated routine contact with soil, dust, and yard debris, and his wife said he regularly ate lunch at job sites without washing his hands. The patient was an avid hunter and had consumed bear meat 3 months before symptom onset.

Based on the patient’s exposure history, we considered infection with *Baylisascaris*, *Toxocara*, *Trichinella*, *Coccidioides*, or other microbial pathogens (Table 1). Because of the patient’s rapid neurologic decline, we initiated albendazole (20 mg/kg/d, given in doses every 12 h) and dexamethasone (4 mg every 6 h) on day 17 for empiric treatment of baylisascariasis or other helminth infection; we also initiated empiric fluconazole and doxycycline. His neurologic symptoms stabilized 1 week later. 

On day 17, serum and CSF samples were sent to the Centers for Disease Control and Prevention (Atlanta, GA, USA) for *Baylisascaris procyonis* immunoassay testing. This test uses a recombinant BpRAG1 antigen and has a sensitivity of 88% and specificity of 98% (*5*). Thirteen days later, the results showed *B. procyonis* antibodies in the serum and CSF samples; results for all other studies were negative (Table 1). Repeat brain MRI on day 29 showed progression of white matter hyperintensity, near complete resolution of enhancement, and mild atrophy (Figure 2, panels E–H). The patient began to show slow, but tangible, improvement in neurologic function after 4 weeks on albendazole and dexamethasone. This combination was continued for 6 weeks, after which albendazole was stopped and a 12-week dexamethasone taper was initiated. By 3 months, the patient had recovered orientation to person and place and limited motor coordination. After 7 months, he could walk with assistance, engage in simple conversations, and perform basic activities of daily living. At that time, CSF showed normalized cell counts (Figure 1).

Most *B. procyonis* roundworm infections occur in young children because their frequency of oral exploration predisposes children to ingestion of infective eggs (*1*–*6*). However, *B. procyonis* infections have been reported in 3 adults and 2 teenagers (*7*–*11*). Of note, those 5 patients had preexisting neuropsychiatric conditions that predisposed them to ingestion of infective eggs via geophagic pica (Table 2) (*7*–*11*). In the case we report, the patient had no predisposing condition, but he probably had occupational exposure, potentiated by insufficient hand hygiene, to raccoon feces.

**Table 2:** Cases of cerebrospinal fluid infection with *Baylisascaris* spp. roundworms in adults and adolescents, United States and Canada, 1986–2015

Year	Patient age, y	Location	Risk factor(s)	Treatment	Outcome	Reference
1986	21	Oregon, USA	Developmental delay and geophagia	Not recorded	Persistent residual deficits	(*7*)
2000	17	California, USA	Developmental delay and geophagia	Albendazole and antiinflammatory drugs	Died	(*8*)
2007	17	Oregon, USA	Altered mentation from drug abuse	None	Aphasia and memory deficits	(*9*)
2009	54	Missouri, USA	Intellectual disability; eating food scraps from public garbage cans	None	Died	(*10*)
2012	73	British Columbia, Canada	Dementia	None	Identified at time of autopsy	(*11*)
2015	63	California, USA	Home or occupational exposure	Albendazole (20 mg/kg/d) + dexamethasone (1 mg/kg/d)	Partial recovery after 6 weeks	This report

Most symptomatic cases of neural larva migrans caused by infection with *Baylisascaris* roundworms have resulted in irreversible neurologic damage, and 5 deaths have been reported (*1*–*3*). Partial to complete recovery occurred in 4 cases, presumably due to a low level of infection at the time of diagnosis, early aggressive treatment, or both (*9*,*12*–*14*).

Because the differential diagnosis for eosinophilic meningitis is relatively restricted, we principally considered infectious etiologies consistent with the patient’s demographics and exposure history. His risk factors associated with an infectious etiology included living and working near a region where *Coccidiodes immitis* is endemic and exposure to raccoon-associated *Baylisascaris* roundworms. For this patient, MRI findings similar to those for other *B. procyonis*–infected patients included subcortical nodular enhancement and linear hyperintensities in the cerebellar white matter on T1- and T2-weighted images (Figure 2) (*14*).

The optimal treatment for baylisascariasis in adults is not known; the current recommendation for albendazole (25–50 mg/kg/d) comes from successful empiric regimens used in children (http://www.cdc.gov/parasites/baylisascaris/health_professionals/index.html#tx). Albendazole is the cornerstone of therapy for *B. procyonis* neural larva migrans and is combined with a corticosteroid to enhance central nervous system (CNS) penetration and mitigate inflammation-associated tissue necrosis (*3*,*4*,*15*). Due to low CNS penetration, ivermectin is ineffective for treating *B. procyonis* neural larva migrans (*1*,*3*). Despite treatment, outcomes are often poor because extensive CNS inflammatory damage and tissue necrosis usually occurs before diagnosis (*3**,**4**,**6*); thus, early recognition of baylisascariasis and prompt initiation of treatment are essential. Because of concern for adverse side effects, including agranulocytosis and hepatotoxicity, we used a 6-week regimen of albendazole plus dexamethasone. We observed reversal of disease progression and a modest neurocognitive recovery after 3 months.

## Conclusions

This case demonstrates that severe neurologic disease from infection with *B. procyonis* roundworms can develop in otherwise healthy adults with incidental exposures. The patient in this report had no history of overt immune compromise and few concurrent conditions and was generally well until the inadvertent ingestion of occult *B. procyonis* eggs. This case highlights the importance of considering baylisascariasis in all patients with eosinophilic meningitis, and it underscores the importance of obtaining a detailed exposure history, understanding the causes of eosinophilic meningitis, and initiating early aggressive therapy when infection is suspected.
