# Correction: Vol. 22, No. 8

**DOI:** 10.3201/eid2211.C22211

**Published:** 2016-11

**Keywords:** errata, erratum, correction

The name of author Natalie Witek was misspelled in *Baylisascaris procyonis*–Associated Meningoencephalitis in a Previously Healthy Adult, California, USA (C. Langelier et al.). The article has been corrected online (http://wwwnc.cdc.gov/eid/article/22/8/15-1939_article).

